# Decision making in clinical practice

**DOI:** 10.1093/jhps/hnag044

**Published:** 2026-10-10

**Authors:** Richard E Field

**Affiliations:** City St George’s University of London

Next time you find yourself conversing with a chatbot, ask yourself, or ask the chatbot whether its program is ‘rule-based’ or ‘Artificial Intelligence (AI)-driven’. Either way, the chatbot’s programming involves multiple steps to analyse the information you provide. The stage at which the bot decides what happens next is known as the ‘Dialogue Management/Decision Logic’ step. Prior to the introduction of AI-driven bots, trained on large language model (LLM) data, programmes followed rule-based decision trees or flowcharts. Rule-based logic relies on calculations like—if intent = X and entity = Y, go to node Z. While the use of AI-driven bots is increasing, their adoption in healthcare has been slower than many areas of our lives because errors carry legal, safety, and clinical consequences [1, 2]. While LLM AI systems offer greater flexibility the healthcare sector continues to rely on rule-based systems. Rule-based systems ensure that every path the conversation can take is known in advance, testable, and traceable. Such systems must be compliant with regulatory authorities [Health Insurance Portability and Accountability Act in the United States, General Data Protection Regulation (GDPR)/United Kingdom (UK) GDPR + National Health Service Digital standards in the UK] and clinical governance sign-off. While a hospital’s compliance team can review a flowchart, it is almost impossible to approve an AI-driven ‘black-box’ decision making process.

In the decades before the advent of bots, flow-charts and protocols were widely adopted throughout healthcare organizations. These tools are auditable and ensure consistency in service delivery. In common with rule-based bots, they enable less experienced staff to follow decision trees to diagnose and treat problems with relatively little or infrequent previous experience of a clinical problem. In contrast, clinicians with years of experience, in a particular area of clinical practice, may exhibit less enthusiasm for such protocols. One interpretation of this difference is to liken the highly experienced clinician to an AI-bot with access to a large dataset of information that enables them to be more agile, and flexible in their decision making. However, the success of the latter approach does depend upon the clinician possessing sufficient relevant experience and like the AI-driven bot, their ‘black box’ reasoning may be harder to audit when they advocate deviations from agreed protocols.

Another example of the difference between rule-based and ‘black-box’ decision making is the tension between evidence-based practice and the adoption of consensus opinion based on subjective clinical experience. In this issue, we present the 2024 International Society for Hip Preservation Physiotherapy agreement on post-operative rehabilitation following hip arthroscopy for femoroacetabular impingement syndrome [3]. The consensus is provided by an expert panel of 11 physiotherapists and 8 surgeons specialising in the management of hip related problems. The panel bring combined clinical experience of 285 and 204 years, respectively. They have acquired their knowledge from tens of thousands of patient interactions, and their shared experience far exceeds that of even the busiest surgeon or physiotherapist. Over the years, there have been times when I have found myself at odds with strongly opinionated and sometimes charismatic individuals who have refused to guide my patients through a path that closely matches that advocated by the consensus panel. My life would have been so much easier if I had been able to share this paper with such individuals and we hope that it proves equally useful to you.

Many of my hip preservation patients ask advice on which physical therapist would best help them rehabilitate after their surgery and what exercises they should work on. Some years ago, a physical therapist, specialising in hip rehabilitation, kindly helped me develop a set of exercises that patients could share with their therapists. While this approach has helped many people, it is always essential to explain that these are only provided as general guidance and that a good therapist will monitor the patient’s progress, identify where they are doing well and where they at progressing more slowly. Every patient’s needs change over time and it is the physical therapist who should adjust any recovery protocol to the individual patient’s needs.

While a consensus protocol developed by a group with deep knowledge of their subject is valuable, it does have inherent limitations. In real world clinical practice, only a small minority of surgeons and physiotherapists care for young, elite athletes. The majority care for patients who have never experienced the challenges of intensive physical training. Therefore, flexibility in the timing of recovery milestones and goals is essential. Also, protocol driven care may provide a reproducible baseline against which deviations can be discerned by clinicians who see many patients undergoing similar treatment. But, clinicians who are less familiar with a specific treatment pathway may become over-focused on ‘the protocol’ and find it more difficult to recognize clinically relevant symptoms or physical signs indicating that a formal clinical review or treatment change may be appropriate.

A further challenge of consensus or protocol driven care is that the clinicians fail to challenge established dogma. One of the greatest advances in hip preservation surgery has been the development of the peri-acetabular osteotomy (PAO) [4] and the evolution of strategies to undertake this procedure with less soft tissue disturbance and less visible scaring [5]. As with any new treatment, PAO surgeons have been obliged to focus much of their attention on proving the clinical [6] and economic [7] value of their interventions. This has obliged surgeons to seek patients for whom there is the greatest chance of success. Numerous publications have identified that the outcome of PAO is adversely affected by increasing patient age and progression of degenerative changes [6, 8, 9]. It may be speculated that fear of being identified as a surgeon whose interventions achieve poor or sub-optimal outcomes may have led some surgeons to minimize their PAO surgery on patients in their thirties and forties. In this issue, we present a paper from Fischer and colleagues [10] in Greifswald showing that favourable mid-term outcomes can be achieved when PAO is undertaken on patients in their thirties and forties. We hope that this paper will provide the wider hip preservation community to further explore the challenging mid-ground where it is most difficult to advise a patient whether they would be best served by hip preservation surgery or joint replacement.

Finally, the relative value of evidence-based treatment against current consensus relies heavily on our confidence in the strength of the evidence or experience provided by each approach. Our belief in evidence-based treatment depends, in large part, on our confidence that we are basing our opinion on robust data. In this issue, we present a paper from Cervantes and his colleagues [11], at Rush University exploring the application of what has become known as the ‘Fragility Index’ (FI) or ‘Continuous Fragility Index’ (CFI) to randomized controlled trials comparing hip arthroscopy to conservative management for the treatment of femoroacetabular impingement syndrome. The paper introduces us to the application of an idea first articulated by Feinstein in 1990 [12] and later developed by Walsh [13]. The FI is the minimum number of patients in a trial whose outcome would need to change (from one outcome to an alternative outcome) to flip a statistically significant result to non-significant (*P* ≥ .05) result. Some methodologists argue the Fragility Index is itself flawed because it is mathematically tied to the *P*-value and sample size in predictable ways, and that confidence intervals already convey precision more informatively. It remains to be seen whether the FI and CFI will add to our confidence in our decision making and the advice we provide to our patients.

We hope that you enjoy all the papers in issue 13.3 and look forward to your contributions to the evolving discipline of hip preservation surgery.
